# Circulating Glutamate and Taurine Levels Are Associated with the Generation of Reactive Oxygen Species in Paroxysmal Atrial Fibrillation

**DOI:** 10.1155/2016/7650976

**Published:** 2016-01-06

**Authors:** Shintaro Takano, Kousuke Fujibayashi, Nakaba Fujioka, Ei-ichi Ueno, Minoru Wakasa, Yasuyuki Kawai, Kouji Kajinami

**Affiliations:** Department of Cardiology, Kanazawa Medical University, 1-1 Daigaku, Uchinada 920-0293, Japan

## Abstract

Atrial fibrillation (AF) is the most common cardiac arrhythmia, but its proarrhythmic mechanism remains to be elucidated. Glutamate (Glu) and taurine (Tau) are present in the myocardium at substantially higher concentrations than in the plasma, suggesting their active role in myocardium. Here, we tested the hypothesis that the metabolism of Glu and Tau is altered in association with the generation of reactive oxygen species (ROS) in patients with AF. Fifty patients with paroxysmal AF and 50 control subjects without a history of AF were consecutively enrolled. Circulating Glu and Tau levels were measured and correlations between Glu/Tau and ROS levels were examined. Glu/Tau content was significantly higher in patients with AF versus controls (Glu: 79.2 ± 23.9 versus 60.5 ± 25.2 nmol/L; Tau: 78.8 ± 19.8 versus 68.5 ± 20.8 nmol/L; mean ± standard deviation (SD), *p* < 0.001 for both). Glu/Tau levels also showed an independent association with AF by multiple logistic regression analysis. Glu and Tau levels both showed significant positive associations with plasma hydroperoxide concentrations. These data suggest a novel pathophysiological role of Glu and Tau in association with ROS production in paroxysmal AF, providing new insights into the elevated amino acid content in cardiac disease.

## 1. Introduction

Atrial fibrillation (AF) is the most common cardiac arrhythmia encountered in clinical practice. While several electrophysiological mechanisms have been proposed to underlie the initiation and perpetuation of AF, the cellular proarrhythmic mechanism of the disorder remains unknown. The *α*-amino acid, glutamate (Glu), and the *β*-amino acid, taurine (Tau), are both normally present in the myocardium at substantially higher concentrations than in the plasma and in this way differ from other amino acids [1, 2]. Moreover, myocardial Glu/Tau concentrations are significantly altered during ischemia/reperfusion so as to maintain metabolic and ionic homeostasis in cardiomyocytes [3–5]. These findings suggest that Glu and Tau dysregulation may feature in the pathogenesis of AF.

Glutamate plays an important metabolic role in the ischemic myocardium. Deamination of glutamate to *α*-ketoglutarate provides an alternative pathway for entry of substrate into the tricarboxylic acid cycle [6, 7]. This conversion of Glu to *α*-ketoglutarate and the following 2 reactions of Krebs' cycle could yield GTP under hypoxia/ischemia condition. It has also been reported that myocardial ischemia decreases intracellular concentrations of Glu and ATP [8], whereas Glu loading stimulates metabolic flux and improves cell recovery after hypoxia in isolated cardiomyocytes [9]. These previous observations regarding the role of Glu in myocardial metabolism might be applied to the conditions of demand-supply imbalance as observed with AF. Glu receptors (GluRs) are found in various peripheral tissues, including in the myocardium. In the human heart, the N-methyl-D-aspartate receptor (NMDA-R), a subtype of GluR as determined by selective agonism of NMDA, is mainly localized in nerve terminals, ganglia, conducting fibers, and atrial myocytes [10]. Recent work indicates that NMDA-R activation induces calcium mobilization and its overload, triggers mitochondrial membrane potential depolarization, stimulates the generation of reactive oxygen species (ROS), and provokes apoptosis in cardiomyocytes [11].

Tau, for its part, participates in many diverse biological and physiological functions, embracing osmoregulation, modulation of ion transport, and antioxidation. Many of these actions within the cardiovascular system seem to participate in the maintenance of intracellular calcium homeostasis [12, 13]. An atrial tissue study indicated that tachycardia-induced calcium overload of cardiomyocytes substantially shortens the refractory period, resulting in electrical remodeling and diminished atrial contractile function. Tachycardia-induced calcium overload also leads to cellular hypertrophy and mitochondrial dysfunction, and potentially to ROS production as well [14, 15]. The atrial effective refractory period progressively shortens after the onset of AF, and rapid atrial activation triggers intracellular calcium overload in atrial cells and complex changes in intracellular calcium handling/contractile remodeling [16]. The changes in atrial cell calcium handling might then contribute to both the initiation and perpetuation of AF; alternatively, calcium metabolism might deteriorate subsequent to AF [17–19].

Based on the above experimental evidence, we hypothesized that patients with AF would exhibit altered amino acid metabolism in the myocardium, especially regarding Glu and Tau content, in association with ROS generation stemming from mitochondrial dysfunction. The present study evaluated this hypothesis by investigating plasma Glu, Tau, and ROS levels in patients with paroxysmal AF.

## 2. Materials and Methods

### 2.1. Subjects

Study subjects (*n* = 100) were enrolled from 160 consecutive patients who were admitted for scheduled radiofrequency catheter ablation (RFCA) or electrophysiological study (EPS) at Kanazawa Medical University Hospital (Uchinada, Japan) between May 2011 and September 2012. Patient and control groups consisted of 50 subjects each (see below). Patients undergoing hemodialysis and those with structural heart disease (e.g., valvular heart disease, congenital heart disease, dilated or hypertrophic cardiomyopathy, or congestive heart failure) were excluded from the study. Patients who previously received or had a suspected diagnosis of atrioventricular block or sick sinus syndrome with a heart rate of <40 beats per minute were also excluded.

Hypertension was defined herein as a systolic blood pressure of ≥140 mmHg, a diastolic blood pressure of ≥90 mmHg, and/or the use of antihypertensive medications. Hyperlipidemia was defined as a total cholesterol level of ≥220 mg/dL, a fasting triglyceride level of ≥150 mg/dL, a high-density lipoprotein cholesterol level of <40 mg/dL, and/or the use of lipid-modifying medications. Diabetes mellitus was defined as a fasting plasma glucose level of ≥126 mg/dL, a hemoglobin (Hb) A1c content of ≥6.5%, and/or the use of antidiabetic medications. Coronary artery disease was defined as an angiographically proven coronary luminal narrowing of >50% in at least one of three major vessels. The estimated glomerular filtration rate (eGFR) for each patient was calculated based on the patient's age and serum creatinine level by using the equation recommended by the Japanese Society of Nephrology [20]. Paroxysmal AF was defined as a history of one or more episodes of medically or self-terminated AF within a 7-day period before procedure. Ultimately, 50 patients were enrolled who presented with paroxysmal AF (the AF group) and 50 age-matched patients with normal cardiac function and without a history of AF or atrial tachycardia (the control group). The study complied with the Declaration of Helsinki, and the study protocol was approved by the Ethics Committee of Kanazawa Medical University (Uchinada, Japan), and all patients gave their written informed consent prior to study enrollment.

### 2.2. Blood Sample Measurements

All participants fasted for at least 8 h prior to the initiation of the study. Just before the RFCA or EPS procedure, blood samples were collected from the aortic root and transferred into a vacuum blood collection tube containing EDTA-2Na. Plasma was separated by centrifugation (2500 ×g) at 4°C for 10 min and stored at −80°C until use. Quantitative determination of Glu and Tau levels was performed by liquid chromatography/mass spectrometry analysis. Intra-assay and interassay variability (CV) values for Tau and Glu measurements were all <5.0% over these different amino acid concentrations (*n* = 10). To evaluate plasma ROS levels, the diacron reactive oxygen metabolites (dROM) test was performed by using a FRAS4 System (H&D, Parma, Italy). The dROM test is a colorimetric assay for the assessment of hydroperoxide levels. Briefly, hydroperoxide molecules are converted into radicals that oxidize N,N-diethyl-para-phenylendiamine. The oxidized product is then spectrophotometrically detected at 505 nm. The units of this measurement are expressed as Ucarr (Carratelli unit) values, where one unit corresponds to 0.8 mg/L hydrogen peroxide. Based on an earlier study carried out among the healthy population, the normal hydroperoxide range is 250–300 Ucarr [21], and data regarding assay validity have previously been reported [21, 22]. Other laboratory measurements (described in Table 1) were obtained by using standard methods for overnight fasting blood samples.

### 2.3. Statistical Analyses

Continuous variables are expressed as the means ± the standard deviation (SD), unless otherwise noted. Categorical variables are expressed as absolute numbers with percentages. For continuous variables, differences between two groups were evaluated by using an unpaired *t*-test or the Mann-Whitney *U* test, depending on the distribution of the data (normal or not normal). Categorical variables corresponding to each patient's clinical background were compared by using the chi-squared test. Correlations between two variables were assessed by calculating the Spearman rank correlation coefficient. To examine the relationship between AF presentation and demographic, echocardiographic, and laboratory variables, including Glu and Tau levels, a univariate logistic regression analysis was initially performed, followed by a stepwise multivariate logistic regression analysis. Age, gender (male = 1, female = 2), body mass index, hypertension (absence = 1, presence = 2), left ventricular ejection fraction, left atrial diameter, eGFR, total cholesterol, HbA1c content, and aortic Glu or Tau levels for multivariate logistic regression analysis model 1 or model 2, respectively, were employed as initial independent variables. The similar analyses were performed to examine the relationship between ROS levels and aforementioned variables by means of univariate and multivariate linear regression analyses. Data analyses were conducted by using Statflex software, version 6.0 (Artech Co., Ltd., Japan). All statistical tests were two-sided, and *p* values of < 0.05 were considered statistically significant.

## 3. Results

Baseline characteristics of the study subjects are shown in Table 1. Despite our efforts to match all variables between AF patients and control subjects, significant differences remained in gender distribution and the prevalence of hypertension, with a larger number of male subjects and a greater prevalence of hypertension in the AF group.

The Glu/Tau levels of the AF group were significantly higher than those of the control group (Table 2). Hydroperoxide levels of the AF were slightly higher than the control group, but the difference failed to reach statistical significance. We next explored the potential determinants of Glu/Tau levels by investigating the association between amino acid content and clinical and laboratory measures in all subjects as a whole. Glu levels showed a significant and positive association with body mass index, while Tau levels showed an association with total cholesterol levels (Table 3). Furthermore, Glu and Tau levels correlated significantly with each other. Gender, hypertension, smoking, or concomitant use of an angiotensin receptor blocker/beta blocker did not significantly impact either Tau/Glu levels (data not shown). Hydroperoxide levels showed significant positive associations with both Glu (*γ*_*s*_ = 0.32, *p* < 0.01) and Tau (*γ*_*s*_ = 0.22, *p* < 0.01) content.

Univariate logistic regression analysis was employed to evaluate the association of AF with clinical and laboratory measures, including Glu and Tau levels. The analysis revealed that male gender, the presence of hypertension, and Glu/Tau levels all showed significant associations with AF (Table 4). Further multiple logistic regression analysis using Glu-inclusive model 1 demonstrated a significant association between Glu content and AF, but not between any of other variables and AF. In addition, utilization of Tau-inclusive model 2 demonstrated significant associations between Tau content and AF, and between male gender and AF (Table 4).

As for the association between ROS levels and these variables, univariate linear regression analysis revealed that age, body mass index, Glu, and Tau all showed significant association with ROS levels (Table 5). Multivariate linear regression analysis using Glu-inclusive model 1 demonstrated that ROS levels were significantly and independently associated with age, body mass index, and aortic Glu content. As for utilization of Tau-inclusive model 2, ROS levels were significantly associated again with age, and with body mass index and Tau content with borderline significance (Table 5).

## 4. Discussion

The present study demonstrated that plasma levels of Glu and Tau in patients with paroxysmal AF were significantly higher than those in control subjects. Furthermore, multiple logistic regression analysis revealed that aortic Tau and Glu levels were independently associated with AF. Glu and Tau levels both showed positive and significant associations with the concentration of a key ROS in the plasma, hydroperoxide.

To our knowledge, this is the first clinical study to investigate the relationship between plasma Glu and Tau content in association with paroxysmal AF. Tau is primarily synthesized from cysteine, which is itself derived in part from methionine in the presence of vitamin B6. In healthy human subjects, plasma Tau levels do not vary greatly with the availability of dietary Tau due to Tau reabsorption in the proximal tubule. Moreover, dietary Glu/Tau content does not parallel that of total amino acid availability, and orally ingested Glu/Tau levels generally do not affect Glu/Tau plasma levels, because both amino acids are utilized in the intestinal tract [23]. Therefore, dietary amino acid content is probably not responsible for the increased Glu and Tau levels observed herein in patients with AF. Preliminary data from our group recently indicated that biopsy-proven idiopathic cardiomyopathy patients have a clearly reduced concentration of these two amino acids relative to healthy controls, suggesting that elevated circulating Glu/Tau levels represent a novel characteristic of AF.

One might assume that the current study observations result from selective hemodynamic alterations secondary to AF. However, we found no significant difference in Glu and Tau levels between blood samples drawn from patients in AF rhythm (mean 72.9 nmol/L, *n* = 4) and our subjects with paroxysmal AF in sinus rhythm. Therefore, our data is probably not explained by hemodynamic alterations resulting from AF. In the central nervous system, the enhanced release of Glu and Tau was previously described as the mechanism responsible for metabolic alterations in Glu and Tau content in response to oxidative stress [24]. Positive association of Glu with BMI (Table 3) suggests that Glu might be released from peripheral tissues, especially for skeletal muscle which contains Glu and Tau abundantly. Ischemia or decrease of osmotic pressure has been reported to enhance Glu release from various cells in association with Tau as compensatory to Glu release [25]. Despite the fact that serum osmotic pressure did not show any association with Glu nor Tau in our study (data not shown), alteration of tissue microcirculation caused by AF might produce ischemic condition leading to these amino acids' release. In addition, from this viewpoint, Glu/Tau might be a candidate molecule to play a role in the recently reported novel association between endothelial dysfunction and future AF event [26].

To examine whether ROS content might similarly be a determinant of the increased Glu levels, we investigated the relationship between aortic Glu content and hydroperoxide levels and uncovered a significant positive association between the two variables. This finding might be rationalized by AF-associated mitochondrial calcium overload and ROS generation, thereby prompting the speculation that NMDA receptors are overstimulated to trigger excessive calcium influx. Previous report described NMDA-R activation by Glu could induce cardiac electrical remodeling, which resulted in the susceptibility to ventricular arrhythmias [27]. Hence, myocardial injury via ROS generation may be responsible for the observed association between elevated circulating Glu levels and AF.

Tau reportedly has a high capacity as a free radical scavenger and can modulate angiotensin II-triggered Na^+^-Ca^2+^ exchange and reduce angiotensin II-induced cardiac hypertrophy and arrhythmia [28, 29]. Tau was considered to maintain intracellular calcium homeostasis by various mechanisms, resulting in the modulation of action potential duration [30]. Therefore, increased circulating Tau content in AF patients is hypothetically a physiologically relevant compensatory response to augmented ROS production in AF. As shown in Table 3, aortic Tau levels showed significant and positive association with total cholesterol levels, which was not true for Glu levels. Experimental studies using animal model [31] or cultured cell line [30] suggested that Tau treatment resulted in reductions of circulating apolipoprotein B-containing lipoproteins [31], possibly through reduced secretion from liver [32], and by upregulation of low-density lipoprotein receptor [33] as well. These reports described negative associations of Tau with cholesterol levels after high-fat diet feeding. Contrary to these, our study subjects were almost normal in total and high-density lipoprotein cholesterol levels (Table 1) and showed positive association. Further studies are required to investigate potential role of Tau in cholesterol metabolism. Despite the fact that neutrophils contain significant amount of Tau [34], lack of association between neutrophil count and aortic Tau levels (data not shown) did not support the hypothesis that circulating blood cells are potential source of Tau. Further studies, including basic science experiments addressing intracellular Tau metabolism in detail, are required to clarify the precise mechanism of Tau versus Glu action in AF.

We could not obtain direct evidence suggesting the origin of increased ROS levels, but possible explanations were as follows. Previous studies showed that stimulation of NMDA-R in cardiomyocytes leads to increased oxidative stress, mitochondrial dysfunction, and inflammatory cytokine production, all of which lead to cell apoptosis [11, 35]. In peripheral vasculature, NMDA-R activation was reported to link neuronal nitric oxide synthase-derived nitric oxide production and ROS generation [36]. Angiotensin II was shown to promote NADPH oxidase-mediated ROS generation, which was considered as a mechanism responsible for atrial myocyte injury in atrial fibrillation [37]. Further clinical and basic studies are required to investigate protective mechanism of Tau including an antagonism of angiotensin II [38].

The current investigation has several clinical implications. First, compounds directed toward modifying Glu or Tau metabolism are prospective antiarrhythmic agents. Indeed, a new chelate coordination compound, Tau magnesium coordination compound (TMCC), was recently synthesized and tested as an antiarrhythmic drug [39]. The antiarrhythmic effects of TMCC were similar to those of amiodarone, as demonstrated in a cesium chloride-induced arrhythmia model. Second, our data are consistent with a previous report indicating that Tau metabolism might be pathophysiologically involved in AF under conditions of excessive thyroid hormone production [40]. Yet another study utilized a Tau transporter knockout mouse model to show that depletion of the intracellular Tau pool via blockade of Tau uptake from the extracellular space resulted in a cardiomyopathic condition [41]. Again, further basic and clinical studies regarding Tau metabolism are required to establish the putative function of this amino acid in the pathogenesis of cardiac arrhythmias and AF.

The present study has several limitations. First, factors related to patient background might have biased our results. For example, the frequency and duration of fibrillation in the AF group could have affected patient hemodynamics, in turn altering myocardial metabolism. However, our preliminary findings showed that aortic Glu/Tau content in subjects with chronic AF did not significantly deviate from that of control subjects (data not shown). Therefore, we assumed that there was no major influence of variability in AF events on our results. Second, the protocols employed in the current investigation could themselves have influenced data interpretation. For instance, the dROM test only assesses hydroperoxide concentration as an indicator of ROS levels, and the possibility exists that hydroperoxide levels might over- or underestimate the levels of other types of ROS. Third, we did not measure the amounts of biomarkers potentially acting as antioxidants and, therefore, we cannot confirm whether the relationship between dROM measurements and Glu or Tau levels indicates a causal association or events secondary to changes in cellular antioxidant defense systems. Fourth, the small sample size of the present study limited the precision of our analyses. Of note, selection bias for control subjects could not be wholly excluded, because the number of female subjects with normal ventricular function who underwent cardiac catheterization was small. Further studies with larger samples are thus essential to confirm the validity of our results.

## 5. Conclusion

The present observations demonstrate that, in the clinical setting, circulating Glu and Tau levels are elevated in patients with paroxysmal AF. Furthermore, the blood levels of ROS as measured by the dROM assay were significantly and positively correlated with aortic Glu/Tau content. These findings suggest a novel pathophysiological role of Glu and Tau in AF and may provide a new insight into the therapeutic implications of altered amino acid content.

## Figures and Tables

**Table 1:** Characteristics of study subjects.

		Paroxysmal AF	Control	*p* value
		(*n* = 50)	(*n* = 50)	
Age, years		64.6 ± 8.3	61.8 ± 11.4	NS
Male, %		36 (72)	23 (46)	<0.05
Body mass index	(kg/m^2^)	23.6 ± 3.4	23.5 ± 3.5	NS
Smoking, *n*	(%)	11 (22)	13 (26)	NS
Hypertension, *n*	(%)	31 (62)	21 (42)	<0.05
Diabetes mellitus, *n*	(%)	6 (12)	6 (12)	NS
Hyperlipidemia, *n*	(%)	12 (24)	14 (28)	NS
Coronary artery disease, *n*	(%)	0 (0)	1 (2)	NS
Laboratory measures				
Hemoglobin A1c	(%)	5.5 ± 0.5	5.5 ± 0.8	NS
Estimated glomerular filtration rate	(mL/min/1.73 m^2^)	67.1 ± 13.0	73.4 ± 15.9	NS
Brain natriuretic peptide	(pg/mL)	33.6 ± 40.7	73.4 ± 15.9	NS
Total cholesterol	(mg/dL)	197 ± 35	201 ± 32	NS
High-density lipoprotein cholesterol	(mg/dL)	54 ± 13	52 ± 11	NS
Triglycerides	(mg/dL)	139 ± 75	131 ± 68	NS
Echocardiographic findings				
Left atrial dimension	(mm)	40.5 ± 5.8	38.1 ± 6.3	NS
Left ventricular end-diastolic diameter	(mm)	46.7 ± 6.1	46.6 ± 4.5	NS
Left ventricular ejection fraction	(%)	65.2 ± 6.9	65.0 ± 7.4	NS

Values are given as the mean ± the standard deviation (SD), or as total number of percentages. AF, atrial fibrillation; NS, not significant.

**Table 2:** Comparison of Glu, Tau, and hydroperoxide concentration in the AO.

	Paroxysmal AF	Control	*p* value
Glutamic acid (nmol/mL)			
Aorta	79.2 ± 23.9	60.5 ± 25.2	<0.001
Taurine (nmol/mL)			
Aorta	78.78 ± 19.8	68.5 ± 20.8	<0.01
Hydroperoxide (Ucarr)			
Aorta	387.2 ± 87.9	374.9 ± 91.5	NS

Values are given as mean ± SD and numbers (percentages). AF; atrial fibrillation.

**Table 3:** Spearman rank correlation coefficients between aortic Glu/Tau content and clinical variables.

	Glu	Tau
Age	−0.09	−0.14
BMI	0.24^*∗*^	0.18
eGFR	0.17	0.11
T-chol	−0.06	0.26^*∗∗*^
BNP	−0.06	−0.10
HbA1c	0.10	0.04
LAD	0.16	0.02
EF	0.06	0.08
Glu		0.57^*∗∗∗*^
Tau	0.57^*∗∗∗*^	
Ucarr	0.32^*∗∗*^	0.22^*∗∗*^

BMI: body mass index, EF: ejection fraction, eGFR: estimated glomerular filtration rate, LAD: left atrial dimension, T-chol: total cholesterol.

^*∗*^ *p* < 0.05,  ^*∗∗*^ *p* < 0.01, ^*∗∗∗*^ *p* < 0.001.

**Table 4:** Univariate and multivariate logistic regression analyses in association with AF.

	Univariate logistic regression			Multivariate logistic regression					
				Model 1			Model 2		
	OR	95% CI	*p* value	OR	95% CI	*p* value	OR	95% CI	*p* value
Age	1.335	0.89–2.00	0.16	1.452	0.90–2.34	0.13	1.455	0.90–2.34	0.12
Gender	2.739	1.20–6.23	0.016	2.193	0.87–5.54	0.10	3.036	1.20–7.68	0.02
BMI	1.027	0.92–1.15	0.64						
HT	2.378	1.06–5.33	0.04	2.141	0.83–5.55	0.12	2.578	1.01–6.61	0.05
EF	1.001	0.95–1.06	0.97						
LAD	1.061	0.99–1.13	0.08						
eGFR	0.977	0.95–1.00	0.12						
T-chol	0.986	0.99–1.01	0.71						
HbA1c	0.627	0.32–1.22	0.17	0.431	0.18–1.06	0.07			
Glu	1.363	1.14–1.63	<0.001	1.375	1.13–1.68	0.002			
Tau	1.286	1.05–1.58	0.017				1.410	1.10–1.80	0.006

CI: confidence interval, OR: odds ratio; other abbreviations are as in Table 3. BMI, EF, LAD, eGFR, and T-chol were subjected to analysis at 1 kg/m^2^, 1%, 1 mm, 1 mL/min/1.73 m^2^, and 1 mg/dL increase, respectively. Age and Glu/Tau were per 10-year and 10-nmol/mL increase, respectively. In the regression analyses, male = 1 and female = 2; absence of hypertension = 1 and presence = 2. Model 1 includes all clinical and laboratory variables in addition to Glu, but not Tau. Model 2 includes all variables in addition to Tau, but not Glu.

**Table 5:** Univariate and multivariate linear regression analyses in association with Ucarr.

	Univariate linear regression			Multivariate linear regression					
				Model 1			Model 2		
	*β*	*t*-value	*p* value	*β*	*t*-value	*p* value	*β*	*t*-value	*p* value
Age	−1.84	2.10	0.039	−1.98	2.25	0.027	−1.98	2.23	0.028
Gender	−5.29	0.29	0.772	−16.55	0.95	0.347	−8.81	0.51	0.611
BMI	6.50	2.61	0.010	5.09	2.07	0.041	4.92	1.95	0.053
HT	15.72	0.87	0.388	15.75	0.88	0.382	21.18	1.18	0.241
EF	0.26	0.20	0.835						
LAD	1.37	0.94	0.349						
eGFR	0.62	0.95	0.342						
T-chol	0.22	0.84	0.403						
HbA1c	16.56	1.23	0.221						
Glu	0.94	2.86	0.005	0.80	2.37	0.020			
Tau	1.10	2.64	0.009				0.82	1.95	0.054

Abbreviations are as in Table 3. BMI, EF, LAD, eGFR, and T-chol were subjected to analysis at 1 kg/m^2^, 1%, 1 mm, 1 mL/min/1.73 m^2^, and 1 mg/dL increase, respectively. Age and Glu/Tau were per 10-year and 10-nmol/mL increase, respectively. In the regression analyses, male = 1 and female = 2; absence of hypertension = 1 and presence = 2. Model 1 includes all clinical and laboratory variables in addition to Glu, but not Tau. Model 2 includes all variables in addition to Tau, but not Glu.
